# A Comprehensive Review of Recent Advances in Artificial Intelligence for Dentistry E-Health

**DOI:** 10.3390/diagnostics13132196

**Published:** 2023-06-28

**Authors:** Imran Shafi, Anum Fatima, Hammad Afzal, Isabel de la Torre Díez, Vivian Lipari, Jose Breñosa, Imran Ashraf

**Affiliations:** 1 College of Electrical and Mechanical Engineering, National University of Sciences and Technology (NUST), Islamabad 44000, Pakistan; imranshafi@ceme.nust.edu.pk; 2 National Centre for Robotics, National University of Sciences and Technology (NUST), Islamabad 44000, Pakistan; anumfatima427@gmail.com; 3 Military College of Signals (MCS), National University of Sciences and Technology (NUST), Islamabad 44000, Pakistan; hammad.afzal@mcs.edu.pk; 4 Department of Signal Theory and Communications and Telematic Engineering, University of Valladolid, Paseo de Belén 15, 47011 Valladolid, Spain; 5 Research Unit in Food Technologies, Agro-Food Industries and Nutrition, Universidad Europea del Atlántico, Isabel Torres 21, 39011 Santander, Spain; vivian.lipari@uneatlantico.es (V.L.); josemanuel.brenosa@uneatlantico.es (J.B.); 6 Research Unit in Food Technologies, Agro-Food Industries and Nutrition, Universidad Internacional Iberoamericana, Campeche 24560, Mexico; 7 Research Unit in Food Technologies, Agro-Food Industries and Nutrition, Fundación Universitaria Internacional de Colombia, Bogotá 111311, Colombia; 8 Universidade Internacional do Cuanza, Cuito EN250, Bié, Angola; 9 Research Unit in Food Technologies, Agro-Food Industries and Nutrition, Universidad Internacional Iberoamericana Arecibo, Puerto Rico, PR 00613, USA; 10 Department of Information and Communication Engineering, Yeungnam University, Gyeongsan 38541, Republic of Korea

**Keywords:** E-health services, healthcare, deep learning, image processing, medical imaging

## Abstract

Artificial intelligence has made substantial progress in medicine. Automated dental imaging interpretation is one of the most prolific areas of research using AI. X-ray and infrared imaging systems have enabled dental clinicians to identify dental diseases since the 1950s. However, the manual process of dental disease assessment is tedious and error-prone when diagnosed by inexperienced dentists. Thus, researchers have employed different advanced computer vision techniques, and machine- and deep-learning models for dental disease diagnoses using X-ray and near-infrared imagery. Despite the notable development of AI in dentistry, certain factors affect the performance of the proposed approaches, including limited data availability, imbalanced classes, and lack of transparency and interpretability. Hence, it is of utmost importance for the research community to formulate suitable approaches, considering the existing challenges and leveraging findings from the existing studies. Based on an extensive literature review, this survey provides a brief overview of X-ray and near-infrared imaging systems. Additionally, a comprehensive insight into challenges faced by researchers in the dental domain has been brought forth in this survey. The article further offers an amalgamative assessment of both performances and methods evaluated on public benchmarks and concludes with ethical considerations and future research avenues.

## 1. Introduction

Due to the outbreak of COVID-19, several countries have been affected, leading to a global emergency. The rise in COVID-19 has brought challenges in maintaining patients’ dental health and providing urgent dental care to mitigate risks of missed diagnosis. Artificial intelligence (AI) has evolved rapidly in terms of complexity, diversity, and computational capabilities, especially in medicine [1,2]. AI has emerged as one of the prospective technologies in healthcare, making significant progress in predictive machine-learning models for dental care [3]. The potential applications of AI apply to dental practices and play a significant role in practice management. Dental clinicians may deploy AI systems as a supplemental tool in providing precise dental diagnoses and planning treatments along with early detection of dental conditions leading to improved patient outcomes [4].

Moreover, different techniques implemented to aid in dental disease diagnosis have led to the introduction of different imaging systems, including X-ray autoradiography (intraoral and extraoral) imaging and near-infrared imaging. X-ray imaging systems have been used widely for pneumonia and COVID-19 detection [5,6]; furthermore, they have become a norm in dentistry for identifying dental lesions, normal and abnormal dental structures, and predicting treatment outcomes. However, a safer method with non-ionizing radiation is also employed by dentists for diagnosing caries and lesions, known as near-infrared imaging. In dentistry, dental ailments are primarily identified using images of the oral cavity each shot from a different angle; a largely manual process. Thus, human inference plays a significant role in analyzing X-ray imagery to recognize dental structures, bone loss, and cavities.

Several automated systems have been explored in recent times for the dental diagnosis process [7,8,9,10,11,12,13]. However, certain issues need to be addressed including limited datasets, class imbalance, limited generalizability, lack of transparency, and interpretability. Another concern is the lack of external validation. Similarly, machine- and deep-learning techniques have not been utilized to their full potential to be integrated into routine dentistry. Additionally, there are certainly ethical considerations that need to be taken into account. Current state-of-the-art approaches still require improvement to tackle the challenges mentioned above. Hence, this survey aims to provide an in-depth analysis of X-ray and near-infrared image-based dental disease diagnosis. Moreover, the identified research gaps and future research directions are discussed thoroughly in the following sections.

Recently, different public datasets have been released, and several machine- and deep-learning-based disease identification frameworks have been introduced. However, the existing surveys [7,8,9,10,11,12,13] lack benchmarking of the existing approaches on public datasets. The surveys also lack a review of other imaging systems, such as near-infrared, and their associated challenges. To our knowledge, no published work presents a comprehensive performance analysis of the approaches on benchmark public datasets.

This article presents a systematic survey of existing literature on clinical applications of AI using X-ray and near-infrared imaging, and discusses the limitations and challenges associated with the application. The main contributions include:

- Discussions related to the problems unique to dental disease diagnosis and the challenges associated with those techniques.
- Propose a taxonomy classifying the existing literature in X-ray and near-infrared imaging, identifying current trends.
- An in-depth analysis of the recently employed dentistry techniques represents a systematic understanding of the advancement within this field.
- Performance analysis of the current approaches on existing benchmark datasets.
- Recommendations and future directions towards the standardization of artificial intelligence in the field of dental medicine.

The materials and methods involved in the study selection process are provided in Section 2. The imaging systems and challenges encountered in processing them for dental diagnosis are briefly explained in Section 3. The public benchmark datasets available for disease diagnosis along with the evaluation metrics are detailed in Section 4. An inclusive account of the machine- and deep-learning approaches for disease diagnosis is provided in Section 5 and Section 6, respectively, while Section 7 focuses on identifying gaps in research alongside future directions. Finally, Section 8 concludes the review.

## 2. Materials and Methods

### 2.1. Protocol

To assess the reporting quality of systematic reviews, the review of the available literature was conducted according to the Preferred Reporting Items for Systematic reviews and Meta-Analysis (PRISMA) guidelines.

### 2.2. Electronic Search Strategy

A comprehensive electronic search for all relevant studies was performed in the database from 2009 until the 30th of June 2022 in Google Scholar, PubMed/MEDLINE, Institute of Electrical and Electronics Engineers (IEEE) Xplore, ScienceDirect, and Scopus. The search shown in Table 1 was based on the PICO (problem, patient, population, indicator, comparison, and outcome) elements. Each database was searched using adapted keywords from the PICO elements. Additionally, a list of abbreviations is provided in Abbreviations to facilitate reading and assimilation of the information presented in this study.

### 2.3. Eligibility Criteria

#### 2.3.1. Inclusion Criteria

- Timeline: manuscripts from the last fourteen years (2009–2022) focused on the application of artificial neural networks, machine learning, and deep learning in dentistry.
- Language: manuscripts that are available in English were included irrespective of country of origin.
- Data and Outcome: studies with proper mention of datasets used along with predictive and measurable outcomes for quantification of the proposed model.

#### 2.3.2. Exclusion Criteria

- Type of Data Used: studies without clear information on data modalities.
- Methodology: studies without sufficient details of computer vision, machine-learning and deep-learning methods, and techniques employed. Language: manuscripts that are available in English were included irrespective of country of origin.
- Outcome: studies that did not report measurable outcomes.

### 2.4. Study Selection and Items Collected

**Figure 1:**
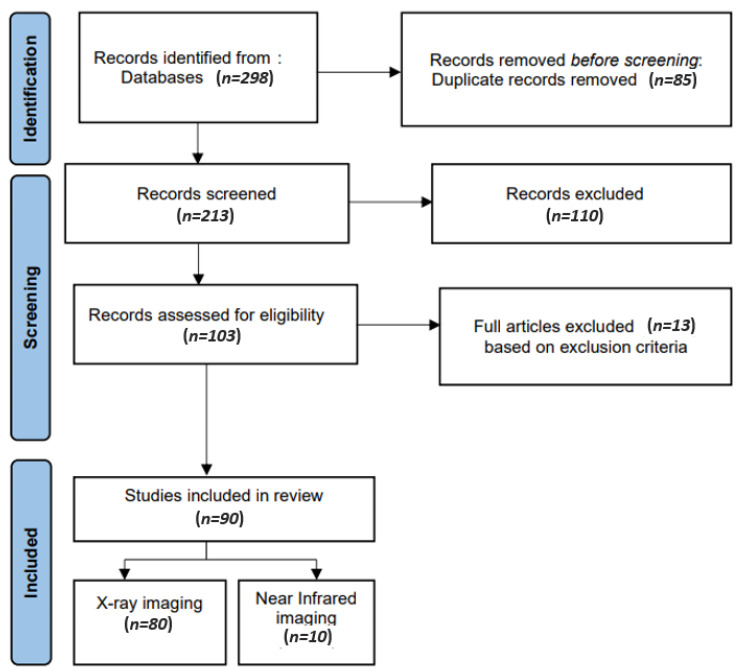
Relevant data about studies included for synthesis.

The title and abstract were screened after removing duplicate papers. The full text of the studies was evaluated based on the eligibility criteria. Finally, the references added to the article were reviewed manually. The full-text papers selected after the screening process are shown in Figure 1.

The following data items were extracted: application, study (author and year), adopted architecture, the task performed, dataset size and dataset split, augmentation, hyperparameters involved for model training, and performance metrics adopted for measuring the performance of the proposed models.

## 3. Imaging Modalities for Dental Disease Diagnosis

**Figure 2:**
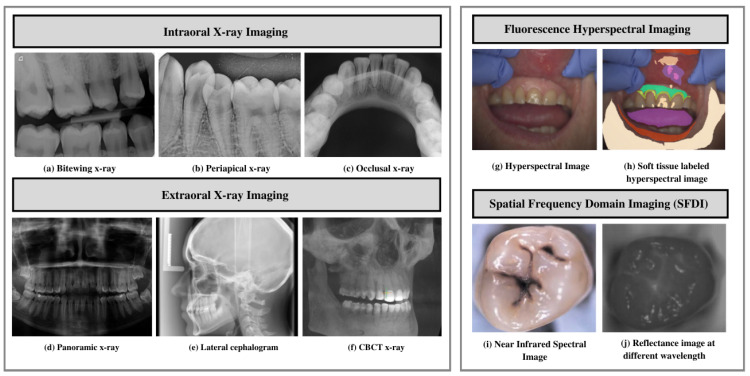
Imaging modalities. (**a**–**c**) Examples of different intraoral imaging. (**d**–**f**) Extraoral imaging modalities. (**g**–**j**) Near-infrared imaging. (**a**) Bitewing X-ray images. (**b**) Periapical X-rays. (**c**) Occlusal X-ray. (**d**) Panoramic X-ray. (**e**) Lateral cephalograms. (**f**) CBCT X-ray. (**g**,**h**) Fluorescence hyperspectral imaging. (**i**,**j**) Spatial frequency domain imaging.

Dental radiography (X-ray) is the most used imaging modality by dentists to identify dental issues such as lesions [14,15,16,17,18,19,20], periapical pathosis [21], and dental restorations [22], and evaluate oral health [23,24,25,26,27]. The examples of imaging modalities employed by researchers for dental disease diagnosis are shown in Figure 2. Different imaging modalities have been explored and their differences are outlined in the following subsections.

### 3.1. X-ray Imaging Systems

To reinstate traditional photographic X-ray films, digital X-ray imaging is employed. X-ray images rely on sensors to produce enhanced images of oral structures [28]. In traditional dental disease diagnosis, the images are evaluated by dentists to identify issues such as tooth lesions and cavities, and devise treatments accordingly [29]. Dentists take several types of dental X-rays to record different mouth views. For example, for the detection of dental cavities, and to monitor mouth and teeth health, intraoral radiographs are used. In addition, dentists use extraoral radiographs to detect impacted teeth, monitor the development and growth of jaws, and identify potential problems in jaws, facial bones, and teeth.

#### 3.1.1. Intraoral X-ray Imaging

Intraoral radiographs remain one of dentistry’s most widely used imaging modalities. These radiographs provide high spatial resolution images that can be used to identify dental and jawbone diseases [30]. Furthermore, these radiographs provide helpful information on bone structure and density. Paralleling and bisecting angles are two techniques to obtain an intraoral radiograph. The sensor is placed on the tooth in parallel planes, leveraging the parallel technique, exposing the radiation. The latter technique involves placing the receptor as close as possible to the tooth and exposing it to a central X-ray beam. The beam is directed perpendicularly to the imaginary line. This line allows bisecting the angle forming a long axis on the tooth and receptor plane [31]. The following are the types of intraoral radiographs used widely by dentists for dental diagnosis and treatment planning.

- Bitewing X-ray provides a detailed account of maxillary and mandibular dental arches in a certain region of supporting bone. Bitewing radiographs aid in detecting tooth decay variations, finding dental decay, and identifying restorations.
- Periapical X-ray portrays teeth in a full-dimensional view of one of either dental arches. The radiograph allows for detecting issues in a specific set of teeth and identifying root structure abnormalities, and detecting the surrounding bone structure.
- Occlusal X-ray shows tooth positioning and their subsequent development in the dental arches of either the maxilla or mandible.

#### 3.1.2. Extraoral X-ray Imaging

Extraoral imaging focuses on detecting dental issues in the jaw and skull. These are generally used to identify problems between the teeth, jaws, and temporomandibular joint.

- On a single radiograph, a panoramic X-ray gives a two-dimensional view of the oral cavity including both the maxilla and mandible. These types of X-rays help identify impacted teeth and diagnose dental tumors [32].
- Lateral cephalogram enables clinicians in tooth examination about the individual jaw profile. Cephalogram imaging shows the entire head’s entire side. This type of imaging aids clinicians in developing treatment plans [33,34].
- Cone-beam computed tomography (CBCT) offers a substantial solution to the conventional radiography demerits. CBCT imaging is used. This type of imaging shows the interior body structures as (three-dimensional) 3-D images and enables identifying fractures and tumors in face bones. This imaging aids surgeons in avoiding after-surgery complications [35].

### 3.2. Near Infrared Imaging Systems

Near-infrared imaging is a nonionizing photo-optical method leveraged for caries detection. This imaging employs long wave radiation against tooth sides [36]. It penetrates objects deeper, thus acquiring good contrast between health and carious tissues [37,38]. This type of imaging offers certain advantages over conventional detection methods, including less radiation exposure. Furthermore, this method provides improved quality images using DIAGNOCAM [36] that transmit light through the alveolar process, including:

- Fluorescence hyperspectral imaging system is a non-contact approach to dental tissue diagnostics. It helps degenerate raw data in a sizeable amount making it suitable for computer vision processing [39]. This imaging system combines spatial and spectral information, enabling dentists to obtain a precise optical characterization of dental issues, including dental plaque. The images are captured using a line scanning camera with 400–1000 nm spectral direction with a 5 nm sampling interval and spatial resolution of 22 μm. In addition, the hyperspectral imaging modality helps assess dental caries severity [40].
- Spatial frequency domain imaging (SFDI) is a quantitative imaging technique [41] that enables the separation of components that are scattered and the optical absorption of a sample. This imaging modality relies on modulating project fringe patterns’ depth at varying frequencies and phases.

### 3.3. Spectral Ranges

There are different spectral bands that have been explored in dental applications.

- Near-infrared, mid-infrared, and long-infrared: These spectral ranges provide valuable information about the chemical composition and molecular structure of dental tissues; this helps in the detection and characterization of dental lesions. Infrared is divided into three spectral regions, mainly near infrared ranging between 4000 and 14,000 cm^−1^, mid-infrared (MIR) ranging between 400 and 4000 cm^−1^, and far infrared, ranging between 25 and 400 cm^−1^ [42].
- Ultraviolet (UV) range: The UV spectral range ranges between 100 and 400 nm wavelength [43]. UV fluorescence techniques have been used to detect caries and also to assess dental materials [44,45].
- Radio frequency (RF) range: Non-ionizing radio frequency pulse with a range of frequencies is used in the presence of a controlled magnetic field for generating MRI [46]. The MRIs generated have found applications in implant dentistry, providing more precise information related to bone density, contour, and bone height [47].

### 3.4. Challenges in Automated Dental Disease Diagnosis

AI-based models have recently gained immense popularity for predicting, detecting, and diagnosing dental diseases. However, specific issues include limited data availability, accessibility, generality, lacking methodological standards, and practical issues revolving around the usefulness and standards in developing such solutions [11]. Therefore, the prime research focus remains on developing efficient and accurate systems to overcome these issues, and help dental practitioners plan treatment and prognosis. Disease classification has been researched extensively. However, certain challenges limit researchers from accomplishing similar levels of achievement in disease segmentation and treatment planning. A few of the open challenges include:

- Limited Data Availability and Comprehensiveness: Due to data protection concerns, medical, especially dental, data is not readily accessible. Moreover, certain challenges including lack in terms of structure and relatively smaller size hinder applications of artificial intelligence techniques [11]. Thus, data availability affects the extent to which deep-learning-based approaches can be employed in this field.
- Data Annotation: Medical data annotation requires specialized knowledge from healthcare professionals. Moreover, data labeling requires an adequate workforce and the process is cost intensive. In the absence of progressive flow and accurately annotated data, deep-learning algorithms cannot make correct interpretations and accurate predictions [48].
- Limited Generalizability: Varying imaging characteristics lead to limited deep-learning model generalizability [49]. The underlying possible generalizability deficits must be elucidated to facilitate the development of improved modeling strategies.
- Class Imbalance: The predominant occurrence of standard samples as compared to abnormal samples leads to class imbalance [50]. The imbalanced data lead to learning bias in the majority class.
- External Validation: Lack of external validation leads to issues in the replication and transparency of AI-based models within dentistry. The community standards for model sharing, benchmarking, and reproducibility must be adhered to [51].
- Interpretability: Lack in terms of interpretability and transparency makes it challenging to predict failures. Interpretability must be ensured to build a proper rapport between technology and humans, and generalize algorithms for specific tasks [8].
- Expertise Gap: The ability to make accurate diagnoses and treatment plans relies on expertise derived from the extensive knowledge and practical experience. AI may not be able to fully replicate the nuanced decision-making that experienced clinicians possess. Bridging the gap between human expertise and AI capabilities poses a significant challenge in automated dental disease diagnosis.
- Sensitivity and Specificity Limitations: Due to variations in image quality and anatomical structures, AI models may have limitations in achieving high sensitivity and specificity.
- Image Interpretation Issues: The overlapping structures and presence of artifacts make interpreting dental images a daunting task. AI models should overcome these challenges to ensure accurate and reliable interpretation of dental images.
- Variations in Pathology Presentation: Dental diseases manifest in different ways. These variations can be in terms of size, shape, or appearance. AI models are required to be able to take into account these variations accurately to provide accurate detection and classification of different pathologies.

## 4. Dataset and Evaluation Metrics

### 4.1. Benchmarks and Datasets

This section explores the datasets used for research in dental disease diagnosis. The summary of characteristics of public benchmarks for dental disease diagnosis is shown in Table 2.

#### 4.1.1. ISBI2015 Grand Challenge Dental Dataset

- Cephalogram Dataset [52] consisting of 400 cephalograms taken from 400 patients. The images were acquired using CRANEX excel ceph machine and are saved in TIFF format. Two experienced doctors evaluated and manually marked 19 landmarks on the images to generate ground truth masks. The size of each image is 1935 × 2400. The goal of this dataset is to enable researchers to make accurate landmark predictions for practical cephalometric analysis.
- Bitewing Radiograph Dataset [53] comprising 120 bitewing radiographic images collected from 120 patients. The dataset includes seven color-coded areas indicating caries using different colors [54,55]. Moreover, images are marked manually after being reviewed by experienced medical doctors. The dataset aims to enable researchers to investigate a suitable automated segmentation method for identifying seven different areas of the tooth.

**Table 2:** Summary of characteristics of public datasets in dental disease diagnosis.

Dataset (Ref)	Dataset Specifications				Research Challenges
	Size and Modality	Disease Category	Format	Other Qualities	
ISBI-2015 grand challenged dental dataset [53]	120 bitewing images 400 cephalograms	Dental caries (enamel, dentin, pulp) Landmark detection	JPEG TIFF	High data variances	Feature extraction and classification, caries detection and landmark identification
Panoramic dental X-ray dataset [56]	2000 panoramic radiographs	Intraosseous mandible lesions	BMP of 2900 × 1250 pixels	A few low-quality images (blurred or malposed)	Mandible segmentation Identification of anatomical structures
UFBA-UESC dental image dataset [57]	1500 panoramic radiographs	Restoration and dental appliance	JPEG of 1991 × 1127 pixels	High data variability and imbalance in terms of number of images and number of pixels per class	Semantic segmentation
Tufts multimodal panoramic X-ray dataset [58]	1000 panoramic radiographs	Tooth abnormalities	Images and ground truth masks: TIFF/JPEG of 840 × 1615	Instance segmentation and numbering. Short textual descriptions of abnormalities present in each radiograph. Gaze plots from eye-tracking data	Image enhancement, tooth segmentation, and abnormality detection
Oral and dental spectral image database (ODSI-db) [59]	316 spectral images with 215 annotation masks	Occlusal surfaces of lower and upper teeth, face surrounding the mouth, and oral mucosa	Multipage TIFF of 1392 × 1040 pixels	Highly imbalanced in terms of number of images and number of pixels per class	Organ segmentation

#### 4.1.2. Panoramic Dental X-ray Dataset [56]

This consists of 2000 panoramic X-ray images obtained from 116 patients. The images are taken with Soredex Cranex D digital X-ray unit. The images cover medical conditions, including healthy, partial, and completely edentulous teeth. Two subsets were generated from the original dataset. In the dataset, the panoramic X-rays are sorted out based on qualitative features, including the vertical distance between the alveolar process, ramus width, the acuteness of the gonial angle, and the inferior mandible.

#### 4.1.3. UFBA-UESC Dental Image Data Set [57]

The benchmark consists of 1500 panoramic radiographs distributed among ten categories. The categories indicate the presence or absence of dental restorations and appliances from 32 teeth. The other two categories are reserved for implants and patients with more than 32 teeth. The images have been obtained using the X-ray camera model ORTHOPHOS XG 5/XG 5 DS/Ceph from Sirona Dental Systems. This dataset has been valuable to the research community for semantic segmentation.

#### 4.1.4. Tufts Multimodal Panoramic X-ray Dataset [58]

The benchmark comprises 1000 panoramic dental radiography images. The images are labeled by experts to identify tooth abnormalities. The abnormalities are categorized into periapical, odontogenic, pericoronal, inter-radicular, and none. The images are in a generic TIFF/JPEG format. The dataset contains radiographs, labeling masks, gray and quantized maps generated using eye-tracking software, tooth masks with labels, along with a maxillomandibular region-of-interest mask [60]. This dataset can aid in enhancing tooth segmentation algorithms, and allow the incorporation of radiologists’ expertise into creating robust and accurate diagnosis systems.

### 4.2. Evaluation Metrics

Different metrics are used to analyze the dental diagnosis algorithms’ performance. This section provides an overview of the performance metrics and some shared concepts of the initial measures. A summary of the commonly employed performance metrics by most algorithms is given here. The initial measures used for the calculation of metrics include:

- True Positive (*TP*): both the ground truth and method prediction correspond to positive.
- True Negative (*TN*): both the ground truth and method prediction correspond to negative.
- False Positive (*FP*): the ground truth is negative, but method prediction corresponds to positive.
- False Negative (*FN*): the ground truth is positive, but method prediction corresponds to negative.

Accuracy shows the fraction of correct predictions. Precision is the fraction of correct positive predictions on total samples. Specificity indicates the fraction of correct negative predictions. Sensitivity or recall shows the true positive rate (TPR) and is the fraction of correct positive samples over total positive samples. The false positive rate (FPR) is the ratio between negative samples erroneously categorized as positive. The F1 score is the harmonic average between precision and sensitivity. The following equations are used to calculate these metrics

(1) $$\begin{array}{c} Accuracy=\frac{TP+TN}{TP+TN+FP+FN} \end{array}$$

(2) $$\begin{array}{c} Precision=\frac{TP}{TP+FP} \end{array}$$

(3) $$\begin{array}{c} Specificity=\frac{TN}{TN+FP} \end{array}$$

(4) $$\begin{array}{c} Sensitivity=\frac{TF}{TP+FN} \end{array}$$

(5) $$\begin{array}{c} FPr=\frac{FP}{FP+TN} \end{array}$$

(6) $$\begin{array}{c} F1=2\times \frac{Precision\times Recall}{Precision+Recall} \end{array}$$

The ISBI2015 Cephalogram Dataset [53] defined successful detection rate, which indicates the estimated point percentage within each precision range, calculated as shown in Equation (7).

(7) $$\frac{i:||\left(\vec{m}i\right)-\left(\vec{a}i\right)\left|\right|<z}{n}*100\left(\%\right)$$

The receiver operating characteristic (ROC) curve is obtained by plotting TPR against FPR at different thresholds. The area under the curve (AUC) is calculated using ROC, which provides an aggregate performance measure across classification thresholds.

Popular metrics utilized for performance measurement of the employed segmentation algorithm include dice coefficient, also known as the SøRensen dice similarity index, capable of comparing pixel-wise agreement with the corresponding ground truth and segmentation prediction provided by the model [61,62] calculated as shown in Equation (8)

(8) $$\frac{\left(2*TP\right)}{\left(2TP+FP+FN\right)}$$

Another metric is the intersection over union (IoU) also referred to as the Jaccard Index. This particular metric allows measuring the similarity between the prediction and the ground truth and is considered a more precise metric to show accuracy for object segmentation [62].

## 5. Approaches to Dental Disease Diagnosis Using X-ray Imaging

**Figure 3:**
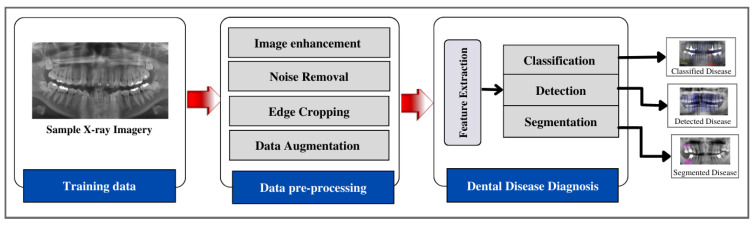
Overview of dental disease diagnosis process flow.

In this section, various research works are discussed to diagnose dental diseases. The overview of the process flow for diagnosing the dental disease is shown in Figure 3. The summary of studies organized by AI application, techniques employed, and the targeted problem is shown in Table 3.

**Figure 4:**
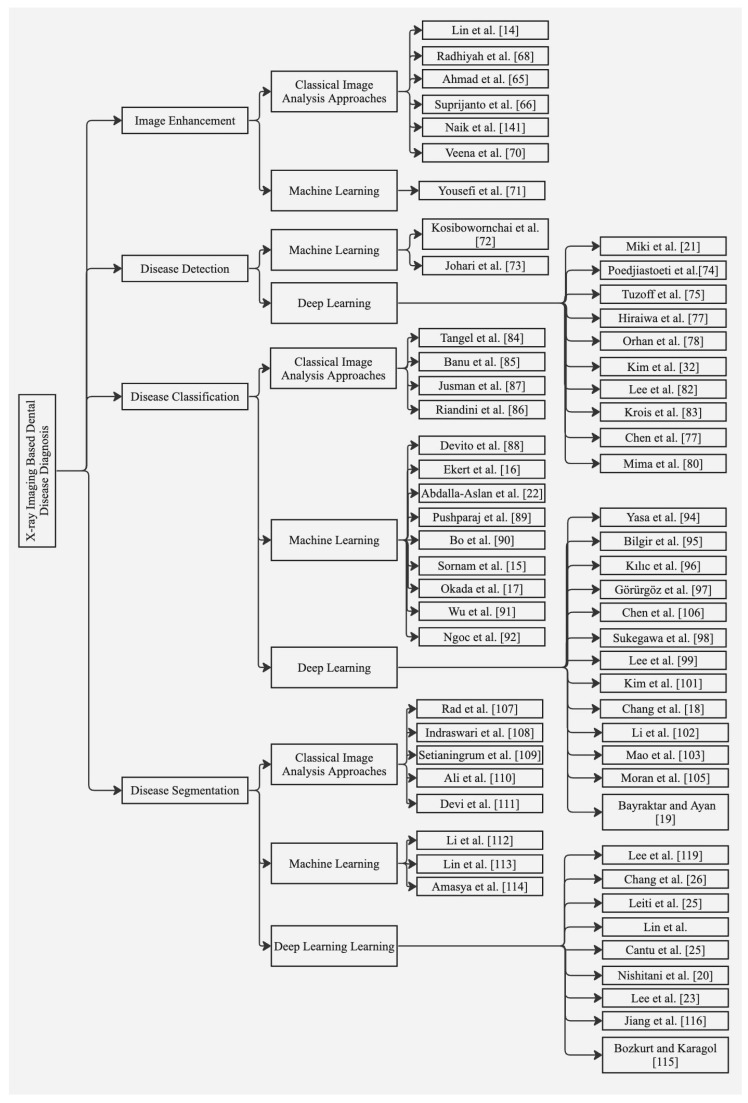
Summary of relevant studies based on classical image analysis, machine- and deep-learning techniques for X-ray imaging.

The presence of residual and topological features makes medical imagery analysis troublesome. The following subsections discuss different artificial intelligence techniques for image enhancement, disease detection, classification, and segmentation. The relevant studies employing classical image analysis, and machine and deep learning for X-ray imaging for dental disease diagnosis are shown in Figure 4.

### 5.1. Image Enhancement

Computer-aided image processing techniques can be employed to improve the contrast and intensity of radiographic images. Lin et al. enhanced X-ray images using a combination of homomorphic filtering and adaptive contrast stretching based on adaptive morphological transformation [14,63]. Ahmed et al. enhanced X-ray image interpretation to improve the diagnostic capability through contrast-limited adaptive histogram equalization (CLAHE). It was found that image enhancement techniques can detect abnormalities with higher efficiency [65]. An improved image enhancement technique based on CLAHE-Rayleigh adopted by Suprijanto et al. provided optimal quality images compared to histogram visualization [66]. Contrast stretching variables have been employed to improve dental radiology image quality [67]. Gaussian filtering and histogram equalization for enhancement of dental radiographs was adopted by Radhiyah and fellow authors [68]. Geetha et al. adopted the Laplacian filter for image sharpening [69]. For the characterization of dental radiographs, Veena et al. adopted contrast adjustment and histogram equalization using panoramic radiographs [70]. Few studies have employed machine learning for X-ray image enhancement in the dental field. However, in one study, Yousefi et al. formulated an image enhancement technique based on wavelet image fusion and a Bayesian classifier [71].

### 5.2. Disease Detection

Leveraging machine learning to diagnose vertical root fractures, a study evaluated neural networks using intraoral digital radiographs and demonstrated their ability to make accurate predictions regarding root fractures and surrounding bone [72]. However, it is important to note that diagnosing root fractures is best performed using CBCT (cone-beam computed tomography) images rather than screening images such as orthopantomographs (OPTs) due to their limited reliability in this context. In line with this, Johari et al. presented a study utilizing a probabilistic neural network (PNN) specifically designed to detect vertical tooth fractures using CBCT images. They found that CBCT images provided more effective diagnostic outcomes compared to periapical radiographs [73]. Therefore, when applying AI algorithms for the diagnosis of root fractures, it is crucial to utilize CBCT images for more accurate and reliable results.

Deep-learning algorithms have been employed significantly for detecting periapical pathosis. Miki et al. investigated an automated method based on a deep convolutional neural network (DCNN) for dental charting using CBCT images yielding an accuracy of 91.0% [21]. For the detection of dental tumors, a study evaluated CNN on ameloblastomas and keratocystic tumors, achieving sensitivity, specificity, and accuracy of 81.8%, 83.3%, and 83%, respectively, based on panoramic X-ray images [74]. In another attempt, Tuzzoff et al. investigated DCNN on panoramic radiographs for tooth numbering and dental charting [75]. A faster RCNN-based method was proposed by Chen et al. for detecting and numbering teeth in periapical radiographs achieving both accuracy and recall of 90% [76]. Hirawa et al. evaluated a deep-learning-based system using panoramic radiographs to assess the number of distal roots present in the mandibular. The system was capable of detecting additional roots yielding encouraging performance [77]. In a study by Orhan et al., a deep convolutional neural network (DCNN) was proposed using CBCT images for tooth detection and numbering specific teeth. The system successfully detected 142 out of 153 periapical lesions with recall, precision, and F1 score of 89%, 95%, and 93%, respectively [78]. Deep learning has been used widely for tooth detection and identification. Chung et al. proposed a point-wise localization and distance regularization method for individual tooth detection. The model was able to localize existing and missing teeth yielding a precision of 99.7% and recall of 97.2% [79]. For automatic tooth region detection, Mima et al. investigated Faster R-CNN, using four cross-validations on panoramic X-ray images. The model could classify 32 tooth types with an accuracy of 91.7% and a mean IoU of 0.748 [80]. Another study explored a single shot multibox detector (SSD) network with a side branch; the model achieved a detection rate of 99.03% and a classification rate of 96.79% on panoramic X-ray images [81]. For detecting periodontal bone loss, Kim et al. proposed DeNTNet, a transfer learning-based deep convolutional neural network on panoramic dental radiographs yields an F1 score of 75%, higher than the average performance of dental clinicians [32]. For detecting and diagnosing dental caries, Lee et al. evaluated deep CNN based on the GoogleNet Inceptionv3 framework and employed transfer learning to make accurate predictions. The model achieved accuracy for pre-molar, molar, and combined tooth models of 89%, 88%, and 82%, respectively [82]. Later, for periodontal bone loss detection, Krois et al. designed a deep CNN network trained on image segments using panoramic radiographs [83].

While deep-learning algorithms have shown promising results in detecting various dental conditions, it is important to note that the diagnosis of certain dental tumors, such as ameloblastomas or keratocysts, remains challenging even for expert clinicians. These conditions typically require a biopsy for a definitive diagnosis. Therefore, it is crucial to highlight that the automated systems mentioned in the previous studies can provide valuable alerts or indications to clinicians, but they are not capable of making a definitive diagnosis, particularly in the field of oral pathology.

### 5.3. Disease Classification

Multiple-fuzzy-attribute-based methods were adopted to analyze each tooth based on area/perimeter and height/width ratio; the teeth were isolated using integral projection, and then features were extracted. The features were then used to classify the teeth using multiple fuzzy attributes [84]. Banu et al., 2014 performed dental cyst classification using texture parameter estimation based on the gray-level co-occurrence matrix (GLCM) approach. The K-means classifier was employed for classification based on estimated parameters [85]. For osteoporosis assessment using thorax X-ray images, feature extraction was performed using GLCM followed by KNN [86]. Another early attempt evaluated two machine-learning algorithms; support vector machine (SVM) and K nearest neighbors (KNN) were used for dental caries classification based on features extracted using the GLCM algorithm [87]. Using machine-learning techniques for diagnosing proximal dental caries, Devito et al. utilized an artificial multilayer perceptron neural network reporting an improvement of about 39.4% in dental caries detection with a ROC curve area of 0.884 [88]. For periapical pathosis using panoramic X-ray images, a tooth numbering and classification system based on feature extraction using projected edge distribution and geometric properties is proposed in [89] to aid forensic odontologists in classifying premolar and molar teeth. For osteoporosis detection, Bo et al. proposed a two-stage SVM model for classification [90]. Another recent attempt [15] for caries detection involved using a backpropagation network with a linearly adaptive particle swarm optimization algorithm. Ekert et al. diagnosed periapical lesions using panoramic radiographs, yielding a specificity of 87% and sensitivity of 65% [16]. Another study utilized cubic SVM to detect and classify dental restoration, achieving a sensitivity of 94% and classification sensitivity of 98% [22]. Jusman et al. evaluated fine Gaussian SVM and KNN on X-ray images enhanced using the GLCM algorithm for dental caries detection. It was found that fine Gaussian SVM achieves an accuracy of 95.7% for five dental classes [87]. Using periapical radiographic images, Wu et al. developed a program based on image patch histogram classification. The algorithm’s performance was evaluated with different histogram similarity measures on periapical root data [91]. A recent study explored R-CNN combined with expert knowledge for classifying teeth with and without root canal filling using 1000 periapical X-ray images. The model achieved an overall accuracy of 95.6%, sensitivity of 89.5%, and specificity of 97.9% [92]. Several studies employed CBCT images. Okada et al. evaluated the Adaboost algorithm in combination with linear discriminant analysis (LDA). The model yielded an accuracy of 94.1% and can be used to identify periapical lesions [17]. Using cephalograms, a non-parametric method for identifying sagittal patterns was proposed by Nino-Sandoval et al. [93].

Various studies have evaluated the performance of deep-learning-based networks for diagnosing and classifying lymph node metastases, dental implants, bone loss, periapical periodontitis, and caries. For tooth numbering, Yasa et al. proposed a faster region-based convolutional neural network (R-CNN) for tooth identification and numbering using 109 bitewing X-ray images. The model yielded an F1 score, precision, and sensitivity of 95.15%, 92.93%, and 97.48%, respectively, and correctly numbered 697 teeth from the test dataset [94]. A faster RCNN was employed by Bilgir et al. using 249 panoramic X-ray images. The model achieved precision, sensitivity, and F1 score of 96.52%, 95.59%, and 96.06% [95]. Kılıc et al. presented a faster R-CNN Inceptionv2 model for detecting and numbering deciduous teeth using 421 panoramic X-ray images. The model yielded a sensitivity of 98.04%, a precision of 98.04%, and an F1 score of 96.86% [96]. Another recent study, Görürgöz et al., explored faster RCNN on pre-trained GoogleNet Inceptionv3 CNN for automated tooth numbering and jaw classification and correctly numbered 668 teeth from 156 periapical radiographs with F1 score, precision, and sensitivity of 87.20%, 78.12%, and 98.67%, respectively [97]. Sukegawa et al. evaluated five models to classify dental implants using panoramic X-ray images; among the five models, fine-tuned VGG-16 exhibited the highest implant classification performance [98]. Lee et al. evaluated the deep fine-tuned CNN algorithm GoogleNetInceptionv3 to identify and classify dental implant systems using panoramic and periapical radiographs, achieving an area under the receiver operating characteristic curve of 0.971 [99]. Another recent study incorporated multi-task deep learning to investigate dental treatment stages and categorize dental implants using panoramic images. Compared to five DCNNs (ResNet-18,34,50,101 and 152), the proposed model achieved high classification validity with an area under the curve of 0.999 [100]. For implant fixture classification, a pre-trained You Only Look Once (YOLOv3) pre-trained using transfer learning was evaluated using periapical radiographs, achieving an accuracy of 96.4% [101]. Calculating the amount of radiographic bone loss is time-consuming and labor-intensive. To automate this process, different deep-learning methods have been employed. For example, one study evaluated a deep machine-learning algorithm for alveolar bone loss detection yielding an accuracy of 87%, and sensitivity and specificity of 86% and 88%, respectively, using periapical X-ray images obtained from 236 patients [18]. In recent years, deep learning has been used sparsely in classifying common chronic diseases such as periapical periodontitis and caries. Li et al. proposed pre-trained AlexNet which transfers learning using periapical X-ray images for apical lesions. They achieved an accuracy of 91.67% in classifying healthy and unhealthy classes [102]. Chen et al. proposed a fast RCNN to detect dental decay, periapical periodontitis, and periodontitis using periapical radiographs. Lesions were detected with a precision of 50% and recall of 60% for disease [106]. A study by Mao et al. proposed a caries and lesion area analysis model for identifying caries and restorations using bitewing images. Compared to four CNNs (AlexNet, GoogleNet, VGG19, and ResNet50) the model achieved an accuracy of 95.5% for restoration and 90.3% for caries [103]. For apical lesion detection, a study investigated a modified deep-learning model evaluated on 4129 periapical X-ray images yielding an F1 score of 82.9% for dental caries and 82.8% for periapical periodontitis [104]. For approximal caries detection, Moran et al. evaluated the inception CNN-based model using 112 bitewing X-ray images to classify caries based on lesion severity achieving an accuracy of 73.3% [105]. Bayraktar and Ayan trained a real-time object localization and classification YOLO-based CNN model to detect approximal dental caries using 800 bitewing images, achieving accuracy above 90% [19].

### 5.4. Disease Segmentation

Based on classical image analysis approaches, Rad et al. employed the level set method and texture feature segmentation for the segmentation of enhanced images [107]. Decimation-free directional filter bank (DDFBT) and multistage adaptive thresholding were used in [108] to segment teeth based on three main steps. In the first step, the vertical and horizontal directional images are formed. The second step involves noise removal and image enhancement for tooth edge reinforcement. Finally, the tooth is segmented using multistage adaptive thresholding (MAT). Although, for the identification of tooth decay, the Otsu method was employed for automatically setting threshold values without human intervention, it was found that images with image enhancement demonstrated larger threshold values and can be utilized by researchers for discriminant analysis [109]. To improve disease pattern recognition, Ali et al. proposed a fuzzy clustering algorithm based on neutrosophic orthogonal matrices for segmentation. Experiments on real datasets affirmed that the fuzzy clustering algorithm outperforms Otsu in practical applications [110]. For fully automatic hybrid multi-lesion classification, thresholding was performed based on fuzzy membership function for pixels and local information of neighboring pixels for enhanced segmentation for dental cyst delineation [111].

Using machine learning, Li et al. investigated the strengths of machine learning and variational level set methods for fast clinical segmentation. The approach is divided into learning and segmentation using SVM and principal component analysis on both 2D and 3D CT scans and X-ray images [112]. For bone loss and tooth decay detection, Lin et al. evaluated SVM to characterize normal and abnormal regions using variational level set function on periapical radiographs [113]. For diagnosing dental caries using dental radiographs, adaptive threshold and morphological operations followed by SVM have been employed. It was found that the proposed method provides reliable decision support with an accuracy of 96.88%, a sensitivity of 100%, a specificity of 86.6%, and a precision of 96.08% [69]. In addition, a method based on a multisource integration framework to assess maxillary structure variation using CBCT images was employed, yielding a dice ratio of 0.80 [114].

Using deep learning, a mask region-based deep CNN for automated tooth segmentation using panoramic radiographic images was proposed by Lee et al. The model detected localized tooth structures with an F1 score of 87.5% and a mean IoU of 0.877 [23]. Another study by Leite et al. explored two deep-learning models for identifying molars and premolars. The model achieved a sensitivity of 98.9% and a precision of 99.6% [24]. Finally, Cantu et al. evaluated a U-Net-based segmentation network using 3686 bitewing X-ray images yielding an overall accuracy of 80% [25]. Teeth are frequently used for the identification of degraded and fragmented human remains in the event of natural disasters. A study by Bozkurt et al. employed a meta-heuristic optimization-based model for identifying teeth and jaw using 20 panoramic X-ray images. The model achieved an average accuracy of 90.73% for separating the mandibular jaw and maxillary jaw teeth [115]. For diagnosing early lesions, Nishitani et al. proposed U-Net with loss function weighted on tooth edge using 162 panoramic images exhibiting dice index of 0.927 higher compared to U-Net with conventional loss function [20]. In addition, a pre-trained Cifar10Net CNN network was proposed by Lin et al. for the classification and segmentation of proximal caries at different severity levels on periapical X-ray images. The model achieved an area under the curve of 0.805 for image extraction, 0.860 for edge extraction, and 0.549 for image segmentation [14]. To measure radiographic alveolar bone level and assess stages using periapical X-ray images, Chang et al. proposed a deep hybrid method for the detection and classification of periodontal loss of individual teeth, using modified Mask-RCNN based on feature pyramid network (FPN) and ResNet101 as a backbone for preprocessing panoramic X-ray images [26]. In a recent attempt, Jiang et al. employed a two-stage deep-learning architecture based on UNet and YOLO-v4 for tooth localization using panoramic X-rays [116]. The model achieved a classification accuracy of 77% and provided more accurate predictions than dental practitioners. Another study by Lee et al. explored U-Net with ResNet-34. The model achieved an accuracy of 85% for bone area segmentation and tooth segmentation [27].

### 5.5. Benchmarking of X-ray Based Dental Disease Diagnosis Approaches

Most of the approaches proposed for dental disease diagnosis have been evaluated on private datasets, making comparative analysis unfeasible. This section provides a comprehensive comparison of different approaches to public benchmarks in terms of experimental protocols adopted by the studies shown in Table 4, Table 5 and Table 6 and performance metrics in Table 7, Table 8 and Table 9.

## 6. Approaches to Dental Disease Diagnosis Using n1ear Infrared Imaging Systems

**Figure 5:**
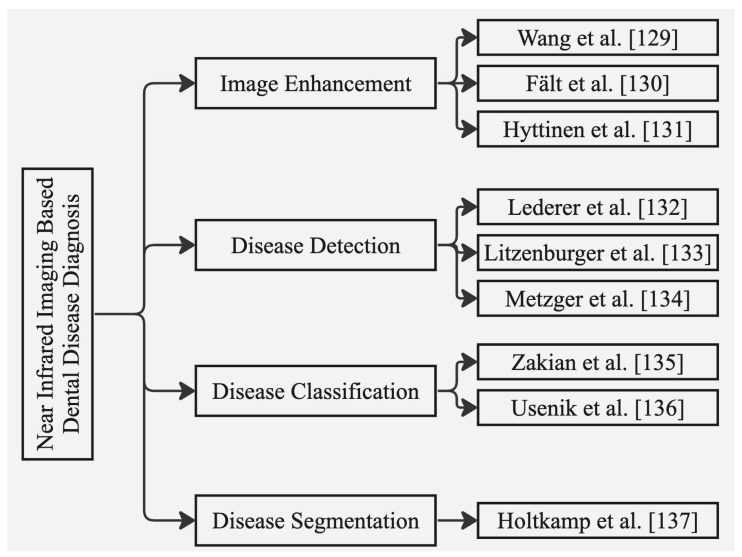
Summary of relevant studies based on classical image analysis, machine- and deep-learning techniques for near-infrared imaging.

NILT imaging is an efficient tool for early, occlusal, proximal, and secondary caries detection and tooth restoration using noninvasive high contrast imaging [36,37,38]. Moreover, NIR spectral features enable reliable identification of dental caries and stages of dental diseases. Few studies have employed NIR imaging to detect dental diseases, as discussed below in detail. To the best of our knowledge, few studies have explored the use of near-infrared imaging for dental disease diagnosis. These studies are mainly based on deep learning for image enhancement, dental disease detection, classification, and segmentation. Figure 5 summarizes the relevant studies based on different computer vision, machine, and deep-learning techniques for dental disease diagnosis using near-infrared imaging. The relevant studies organized by AI application, targeted problem, and imaging type based on near-infrared imaging are shown in Table 10.

### 6.1. Image Enhancement

On spectral reflectance imaging, a multi-spectral imaging technique using various light sources for visualization of oral tissues has been proposed by Wang et al. It was found that spectral analysis increased the efficiency of diagnosis of oral cancer and treatment planning [129]. Another method was proposed by Fält et al. based on particle swarm optimization (PSO). The method improved the contrast between the lesion and non-lesion areas for efficient dental disease diagnosis [130]. As a starting point for spectral image enhancement on a publicly available dataset [59] for dental disease diagnosis, Hyttinen et al. demonstrated contrast enhancement of spectral images using partially negative computational filters derived from principal component analysis (PCA). The study highlighted the use of spectral imaging in treatment planning and preventive care [131].

### 6.2. Disease Detection

Recent studies explored the clinical performances of NIR-based systems for detecting dental caries compared to bitewing radiographs. It was found that near-infrared imaging provides comparable performance for the detection of dental caries compared to bitewing radiographs [132,133,134].

### 6.3. Disease Classification

Zakian et al. employed three spectral bands and a classification model with several user-defined parameters determined empirically. The proposed model lacked in providing consistent and repeatable results [135]. A user parameter-free classification approach was proposed by Usenik et al. involving NIR hyperspectral imaging for early caries detection and classification of healthy and diseased dental tissues. The proposed system was evaluated on 12 extracted human teeth and achieved a sensitivity of 83% and specificity of 99% [136].

To test the generalizability of CNNs on NILT imagery, Holtkamp et al. trained the ResNet classification model on both Vivo and Vitro datasets. The model trained on Vivo and tested on Vitro achieved an accuracy of 70% with an area under the curve of 0.66, and the same model trained on Vitro and tested on Vivo achieved an accuracy of 61% and AUC of 0.60 [137].

### 6.4. Disease Segmentation

Casalegno et al. trained deep CNN on 217 NILT images of occlusal and proximal surfaces. The model achieved an AUC of 85.6% and an overall IoU score of 72.7% for proximal and 83.6% for occlusal lesions [138]. Another study by Schwendicke et al. explored two deep CNNs based on ResNet18 and Resnext50 pre-trained using the ImageNet dataset. In Vivo, 1319 segmented teeth are included, whereas, in Vitro, 226 posterior permanent human teeth are considered. It was found that ResNext50 performed better with regards to performance; the model yielded an AUC of 74% on NILT images of single tooth segments [139].

### 6.5. Benchmarking of Near Infrared Based Dental Disease Diagnosis

Comparative analysis of the methods discussed above is impossible as most of the approaches proposed for near-infrared-based dental diagnosis have been evaluated on private datasets. Hence, in this section, only two studies were retrieved to compare approaches evaluated on the public dataset ODSI-DB [59] in terms of experimental protocols and performance metrics as shown in Table 11.

### 6.6. Assessment of Risk Bias

A comprehensive assessment of bias was performed in this study. Table 12 and Table 13 provide a risk assessment summary for each study, indicating the level of bias based on predefined criteria for both X-ray-based and NILT-based imagery.

## 7. Ethical Considerations and Future Research Directions

Artificial intelligence provides remarkable opportunities for researchers and dental clinicians to diagnose dental diseases. Moreover, this technology offers immense opportunities to advance diagnostics in dentistry. However, due to the lack of publicly available datasets, its adoption has become a difficult challenge. Similarly, using machine and deep learning has not been fully integrated into routine dentistry; data sharing and privacy issues need to be dealt with through federated guidelines. The following subsections highlight the ethical considerations and research gaps, and provide future research directions.

### 7.1. Ethical Considerations

The application of artificial intelligence in dentistry has enormous potential to improve healthcare; however, there are certain ethical issues [141] that need to be addressed to achieve the full potential of AI in dental healthcare. The accessibility and comprehensibility of data are essential aspects of transparency. AI predictions rely heavily on the accuracy of dataset annotations. Poorly labeled data can lead to inaccurate results and limit the efficacy of AI-based dental diagnosis systems [126,142]. Moreover, another ethical aspect indicates using patient data and medical test records to make predictions. The prediction models’ lack of transparency and interpretability remain a limitation due to privacy issues [143]. Other limitations include a lack of systematic conditions such as risk factors [144] and the need for a third party to evaluate treatment outcomes [54].

### 7.2. Research Gaps and Future Research Directions

Artificial intelligence in dentistry is generally not intended to replace dental clinicians. Instead, it helps obtain second-informed opinions based on prediction and mathematical decisions. Although existing approaches are powerful and can be employed for effective and accurate dental disease diagnosis, the research in this domain is still sparse. Certain future directions can pave the way for AI to be used for disease diagnosis and prognosis.

#### 7.2.1. Data Insufficiency

Machine- and deep-learning algorithms require enough data. Imbalances and small datasets remain a barrier to training the models. Very few current studies have addressed the data imbalance problem and utilize small datasets. These studies have applied traditional data augmentation techniques on data to acquire improved performance [117,118,119,124,125]. These techniques lead to data bias and suboptimal performance of trained models. To overcome these issues and deal with insufficient data, few-shot learning [145] can be adopted. Few-shot learning has gained the attention of researchers in medical fields and a few studies have proposed small sample object detection methods based on meta-learning and transfer learning [146].

#### 7.2.2. Class Imbalance Learning

Few studies address the class imbalance problems [131,140] and they are often evaluated on privately available small datasets. Handling imbalanced class distribution is essential to acquiring improved prediction performance. Unreliability in prediction performance occurs due to the capability of the model to learn from the majority class and insensitivity towards the minority class [147]. Hence, different techniques could be applied to balance data, including using a synthetic oversampling technique (SMOTE) [148] that generates data samples by leveraging samples of feature space of the target class combined with features of nearest neighbors. Additionally, generative adversarial networks (GANs) can be applied as they provide additional benefits compared to traditional augmentation techniques. GANs have been used by researchers in the medical field to generate realistic medical images for data augmentation. Moreover, GANs can also be employed to denoise and enhance dental images and generate high-resolution synthetic images for improved detection, classification, and segmentation for more accurate dental diagnosis [149].

#### 7.2.3. Personalized Dental Medicine

Personalized medicine aims to individualize dental care based on the patient’s clinical profile. The linkage of patients’ data gathered from different sources aids in diagnosing dental diseases and developing novel strategies [150]. A personalized approach to managing oral diseases is progressing, including managing chronic orofacial pain and integrating personalized medicine into dental practice. Researchers devised different imaging systems, including radiographic and near-infrared, to distinguish between different dental diseases. To provide effective oral healthcare, certain improvements are needed in validating its routine. Moreover, certain scientific and technological gaps are to be considered, including linkages between clinical outcomes, genotypes, and individual biomarkers, cost-effectiveness, and improved drug design and delivery using artificial intelligence [151]. There is a need for further research in this particular field to develop tools for patient-specific precision healthcare.

#### 7.2.4. Tele-Dentistry

Tele-dentistry has widened the scope of oral healthcare at reasonable costs and helps dentists triage patients needing urgent dental care [152]. It further enables dentists to acquire patients’ medical health records and radiographic images from an online record management system, analyze the data, and provide consultation remotely. Tele-dentistry has several applications, such as telediagnosis, which involves using images to diagnose dental disease remotely, telemonitoring for monitoring dental health, and teleconsultation to provide expert opinion remotely to patients and ward off unnecessary travel for patients facing geographic differences. A certain research gap remains regarding the use of AI in teledentistry. Few studies have explored AI for remote dental diagnosis and monitoring. Studies have been conducted regarding teledentistry in pediatric dentistry [153]. Moreover, teledentistry can prove to be helpful for the differential diagnosis of common lesions [154,155].

#### 7.2.5. Internet of Dental Things (IoDT)

Medicine and dentistry are evolving, and different digital technologies are efflorescing for diagnosing dental diseases and treatment planning. One of the recent smart technology trends is IoDT based on the Internet of Things (IoT) which comprises a network of connected physical gadgets. These devices can be controlled from across the globe using the internet. At present, not many pieces of evidence about IoDT are available. Hence, new research related to IoDT would significantly improve diagnosis and prevent dental diseases. A study by Salagare et al. involved IoDT technology for preventive oral care [156]. In another study, Liu et al. developed a smart dental IoT-based system to provide in-home dental healthcare. The authors leveraged IoT to gather clinical images of patients from ten clinics and applied R-CNN to detect and classify seven dental conditions [157]. To efficiently evaluate patients’ oral health using IoT, Vellappally et al. proposed an IoT based xeno-genetic spiking neural network. The proposed model accurately identified tooth structure, gaps, premolars, and molars, and was helpful in effectively extracting oral features for detecting dental caries, plaque, and periodontal diseases [158]. Sannino et al. investigated IoT-based integrated wireless sensing technology to detect micro displacement to prevent implant failure [159]. IoDT is continuously increasing in dentistry to achieve evidence-based outcomes and improve treatment quality. Further research in this direction can provide more opportunities to provide enhanced dental care.

Additionally, future research is needed in order to combine AI technology not only with diagnostic devices but also with other recently introduced therapeutic features, such as low noise instruments [160] and computerized anesthesia devices (Local anesthesia with SleeperOne S4 computerized device vs traditional syringe and perceived pain in pediatric patients: a randomized clinical trial [161]).

## 8. Conclusions

The progressive development of AI in dentistry has the potential to benefit clinicians and researchers in improving dental care. Current computer vision techniques, machine learning, and deep learning have demonstrated remarkable performance in disease diagnosis and treatment planning, surpassing state-of-the-art approaches. However, it is important to acknowledge that, despite these advancements, AI cannot substitute the invaluable role of human knowledge and experience. The limitations and challenges discussed in this article emphasize the need for human expertise in overcoming complex diagnostic scenarios and making informed treatment decisions. While AI can assist and augment the capabilities of clinicians, it cannot fully replicate the nuanced decision-making process that experienced professionals possess. Therefore, the integration of AI in dentistry should be seen as a complementary tool that enhances clinical practice rather than replacing human experts.

Furthermore, ethical considerations, such as data accessibility, transparency, and privacy, must be carefully addressed to ensure patient trust and protect sensitive information. Standardized protocols and federated guidelines for data sharing and privacy protection are essential in this regard. Keeping in view these ethical considerations, future research directions should focus on addressing data insufficiency and class imbalance issues in AI algorithms. Innovative approaches such as few-shot learning and class imbalance learning can help mitigate these challenges and improve prediction performance. Additionally, personalized dental medicine and tele-dentistry present exciting opportunities for leveraging AI technologies to provide individualized care.

The present study has certain limitations that should be acknowledged. Firstly, the inclusion criteria of the survey focused on studies that employed artificial neural networks, machine learning, and deep-learning techniques in dentistry. Other approaches and techniques that may have been used for disease diagnosis, such as traditional diagnostic methods or emerging technologies, have not been explored in this study. Therefore, the findings may not provide a comprehensive overview of all diagnostic approaches utilized in dentistry. Secondly, the inclusion criteria of the study focused on techniques for dental disease diagnosis based on X-ray imaging and near-infrared imaging techniques. While these modalities are commonly used in dentistry, they represent only a subset of the available diagnostic approaches. The exclusion of other imaging modalities, clinical examination findings, histopathology, or genetic markers may limit the generalizability of the findings and may not reflect the broader landscape of diagnostic approaches in dentistry.

## Figures and Tables

**Table 1:** Description of PICO elements.

Element	Description
Research question	What are the clinical applications and diagnostic performance of artificial intelligence in dentistry?
Population	Dental imagery related to X-ray images (bitewing, periapical, occlusal, panoramic, cephalograms, cone-beam computed tomography (CBCT)) near-infrared light transillumination (NILT) images, fluorescence hyperspectral images, spatial frequency domain images.
Intervention	AI-based models for diagnosis, detection, classification, and segmentation.
Comparison	Different algorithms to predict dental diseases.
Outcome	Measurable and predictive outcomes that include accuracy, specificity, sensitivity, F1 score, intersection over union (IoU), dice coefficient, regression co-efficient receiver operating characteristic curve (ROC), area under the curve (AUC), and successful detection rate (SDR).

**Table 3:** Studies organized by AI application, techniques, and problems targeted based on X-ray imagery.

Application	Technique	Target Problem and Study Number
Image enhancement	Classical image analysis approaches Machine learning	Contrast adjustment [63,64,65,66,67,68,69], image sharpening [70]
		Visibility enhancement [71]
	Deep learning	-
Disease detection	Machine learning	Vertical root fracture [72,73]
	Deep learning	Periapical pathosis [21], dental tumors [74], tooth numbering [75,76,77,78], tooth detection and identification [79,80,81], periodontal bone loss [32,82,83]
Disease classification	Classical image analysis approaches	Tooth detection [84,85], osteoporosis assessment [86], dental caries [87]
	Machine learning	Dental caries [88], proximal dental caries [14], molar and pre-molar teeth [89], osteoporosis [90], dental caries [15], periapical lesions [16,17], dental restorations [22], periapical roots [91], teeth with root [92], sagittal patterns [93]
	Deep learning	Tooth numbering [94,95,96,97,98,99], dental implant stages [100], implant fixture [101], bone loss [18], periapical periodontitis [102,103,104,105], dental decay [106], approximal dental caries [19]
Disease segmentation	Classical image analysis approaches	Feature extraction [107], tooth edge reinforcement [108], tooth decay [109,110], dental cyst delineation [111]
	Machine learning	Bone loss and tooth decay detection [112,113], dental caries [85], assess maxillary structure variation [114]
	Deep learning	Identification of molars and premolars [23,24,25], identification of degraded and fragmented human remains [115], diagnosing early lesions [20], alveolar bone level [26,27], tooth localization [116]

**Table 4:** Comparative analysis of experimental protocols employed by dental disease diagnosis approaches on ISBI-2014–2015 grand challenge dental dataset.

Author, Year (Ref)	Architecture	Task	Dataset Size and Split			Data Augmentation	Hyperparameters		
			Train Set	Valid Set	Test Set		Loss Function	Optimizer	Learning Rate
Zeng et al., 2021 [117]	Three stage cascaded CNN	Landmark detection	150	150	100	Affine transformation	-	Adam	0.001
Song et al., 2020 [118]	CNN with pre-trained ResNet50	Landmark detection	150	150	100	Affine transformation	-	Adam	0.001
Lee et al., 2020 [119]	Bayesian CNN (BCNN)	Landmark detection	150	250	-	Affine transformation	Softmax cross entropy	Adam	0.001
Qian et al., 2019 [120]	Faster R-CNN	Landmark detection	150	150	100	-	Custom loss function	Stochastic gradient descent (SGD)	0.001
Lindner et al. [121]	Random Forest, regression, voting	Landmark detection	150	250	-	-	-	-	-
Ibragimov et al., 2014 [122]	Shape and appearance based landmark refinement with game theory	Landmark detection	150	150	100	-	-	-	-
Chu et al., 2014 [123]	Random forest, regression	Landmark detection	150	150	100	-	-	-	-

**Table 5:** Comparative analysis of experimental protocols employed by dental disease diagnosis approaches on Tufts multimodal panoramic X-ray.

Author, Year (Ref)	Architecture	Task	Dataset Size and Split		Data Augmentation	Hyperparameters		
			Train Set	Test Set		Loss Function	Optimizer	Learning Rate
Pannetta et al., 2022 [60]	UNet with three backbones	Tooth segmentation	85–	150	-	Cross entropy	Adam	0.0001
Nashold et al., 2022 [124]	Multi-objective model	Abnormality detection and localization	900	100	Affine transformation	Binary cross entropy	Adam	0.0001
Karacan et al., 2022 [62]	Tooth segmentation	-	-	-	-	-	-	-

**Table 6:** Comparative analysis of experimental protocols employed by dental disease diagnosis approaches on UFBAUESC dental image data set.

Author, Year (Ref)	Architecture	Task	Dataset Size and Split			Data Augmentation	Hyperparameters		
			Train Set	Valid Set	Test Set		Loss Function	Optimizer	Learning Rate
Yamanakkana et al., 2022 [125]	Two feature aggregation module	Tooth segmentation	1200	150	150	Affine transformation	-	-	-
Chen et al., 2021 [126]	Multiscale structural similarity	Tooth segmentation root boundary extraction	1200	150	150	-	Custom hybrid loss	Adam	0.0001
Zhao et al., 2020 [127]	Two stage attention segmentation network	Tooth segmentation	1200	150	150	-	Custom hybrid loss	Adam	0.001
Kosh et al., 2019 [128]	Ensemble U-Net	Tooth segmentation	1200	-	300	Affine transformation	Cross entropy	Adam	0.0001
Silva et al., 2018 [57]	Mask R-CNN	Tooth segmentation	753	452	295	-	-	-	-

**Table 7:** Comparative analysis of performance metrics of dental disease diagnosis approaches using ISBI cephalometric dataset.

Author, Year (Ref)	Performance Evaluation Metrics							
	Successful Detection Rate (%)							
	2 mm		2.5 mm		3 mm		4 mm	
	Testset1	Testset2	Testset1	Testset2	Testset1	Testset2	Testset1	Testset2
Zeng et al., 2021 [117]	81.3	70.5	89.9	79.5	93.7	86.5	97.8	93.3
Song et al., 2020 [118]	86.4	74.0	91.7	81.3	94.8	87.5	97.8	94.3
Lee et al., 2020 [119]	82.1	82.1	88.6	88.6	92.2	92.2	95.9	95.9
Qian et al., 2019 [120]	82.5	72.4	86.2	76.1	89.3	79.6	90.6	85.9
Lindner et al., 2016 [121]	73.6	66.1	80.2	72.0	85.1	77.6	91.4	87.4
Ibragimov et al., 2014 [122]	71.7	62.7	77.4	70.4	81.9	76.5	88.0	85.1
Chu et al. [123]	39.7	44.1	51.7	57.0	62.1	68.0	77.7	83.8

**Table 8:** Comparative analysis of performance metrics of dental disease diagnosis approaches using Tufts multimodal panoramic X-ray dataset.

Author, Year (Ref)	Task	Performance Evaluation Metrics				
		Accuracy (%)	IoU (%)	Dice Co-Efficient (%)	F1 Score	Recall
Pannetta et al., 2022 [60]	Tooth segmentation (5 categories)	95.01	86.1	91.6	-	-
Nashold et al., 2022 [124]	Abnormality detection and localization (5 categories)	94.9	91.2	-	70.5	-
Karacan et al., 2022 [62]	Tooth segmentation (teeth and maxillomandibular)	-	91.8	95.7	-	-

**Table 9:** Comparative analysis of performance metrics of dental disease diagnosis approaches using UFBA-UESC dental image dataset.

Author, Year (Ref)	Task	Performance Evaluation Metrics					
		Accuracy (%)	Specificity (%)	Precision (%)	F1 Score (%)	Recall (%)	Dice Score (%)
Yamanakkanavar et al., 2022 [125]	Tooth segmentation (10 categories)	97.0	-	-	-	-	-
Chen et al., 2021 [126]	Tooth segmentation and root boundary extraction	97.3	98.45	93.35	-	92.97	93.01
Zhao et al., 2020 [127]	Tooth segmentation (10 categories)	96.94	97.81	94.97	-	93.77	92.7
Koch et al., 2019 [128]	Tooth segmentation (10 categories)	97.2	98.3	92.9	-	-	93.6
Silva et al., 2018 [57]	Tooth segmentation (10 categories)	92.08	96.12	83.73	76.19	79.44	-

**Table 10:** Studies organized by AI application, techniques, and problems targeted based on near-infrared imagery.

Application	Target Problem and Study Number	Image Type
Image enhancement	Contrast enhancement	Spectral reflectance imaging
Disease detection	Dental caries	Near-infrared imaging
Disease classification	Early caries	Near-infrared hyperspectral imaging
Disease segmentation	Proximal and occlusal lesion	Near-infrared transillumination imaging

**Table 11:** Comparative analysis of experimental protocols and performance metrics employed by dental disease diagnosis approaches on ODSI-db.

Ref, Year	Architecture	Task	Dataset Size		Pre-Processing	Hyperparameters			Metric
			Train Set	Test Set		Loss	Optimizer	Epochs	Accuracy
[140], 2021	CenterNet ResNet	Classification and localization (17 categories)	19,215 hyperspectral images	2135	Re-labeled masks using custom algorithm	-	Adam	10,000	62.81%
[131], 2021	Principal component analysis (PCA)	Image enhancement	Spectral images per class	-	Contrast stretching	-	-	-	-

**Table 12:** Studies organized by AI application, techniques, and problems targeted based on X-ray-based imagery.

Application	Technique	Target problem and study number
Image enhancement	Classical image analysis approaches	Contrast adjustment [63,64,65,66,67,68,69] (low), image sharpening [70] (low)
	Machine learning	Visibility enhancement [71] (moderate)
Disease detection	Machine learning	Vertical root fracture [72,73] (low)
	Deep learning	Periapical pathosis [21] (moderate), dental tumors [74] (high), tooth numbering [75,76,77,78] (low), tooth detection and identification [79,80,81] (moderate), periodontal bone loss [32,82,83] (moderate)
Disease classification	Classical image analysis approaches	Tooth detection [84,85] (low), osteoporosis assessment [86] (low), dental caries [87] (low)
	Machine learning	Dental caries [88] (low), proximal dental caries [14] (moderate), molar and pre-molar teeth [73] (low), dental implants [98] (low), dental periapical lesions [17] (moderate)

**Table 13:** Studies organized by AI application, targeted problem, and imaging type based on near-infrared imaging.

Application	Technique	Target problem and study number
Image enhancement	Classical image analysis approaches	Contrast enhancement (low) [129,130,131]
	Machine learning	Spectral image enhancement for dental disease diagnosis (low) [131]
Disease detection	Machine learning	Dental caries detection using NIR imaging (low) [132,133,134]
Disease classification	Classical image analysis approaches	Dental tissue classification using NIR hyperspectral imaging (low) [135,136]
	Deep learning	Dental caries classification using CNNs (moderate) [137]
Disease segmentation	Deep learning	Lesion segmentation using deep CNN (moderate) [138,139]

## Data Availability

Not applicable.

## Abbreviations

The following abbreviations are used in this manuscript:

PRISMA	Preferred Reporting Items for Systematic Reviews and Meta-Analysis
AI	Artificial Intelligence
CNN	Convolutional Neural Network
SVM	Support Vector Machine
DL	Deep Learning
CAD	Computer-Aided Diagnosis
ROI	Region of Interest
CBCT	Cone-Beam Computed Tomography
CNNs	Convolutional Neural Networks
MLP	Multilayer Perceptron
DNN	Deep Neural Network
ANN	Artificial Neural Network
SVMs	Support Vector Machines
RF	Random Forest
KNN	K-Nearest Neighbors
LDA	Linear Discriminant Analysis
PCA	Principal Component Analysis
ROC	Receiver Operating Characteristic
AUC	Area Under the Curve
TP	True Positive
TN	True Negative
FP	False Positive
FN	False Negative
